# What aspects of primary care predict emergency admission rates? A cross sectional study

**DOI:** 10.1186/1472-6963-13-11

**Published:** 2013-01-07

**Authors:** Stephen Gunther, Nick Taub, Stephen Rogers, Richard Baker

**Affiliations:** 1 Public Health Department, NHS Northamptonshire, Northampton, UK; 2 Department of Health Sciences, University of Leicester, Leicester, UK

**Keywords:** Emergency admissions, Primary care, Access, Preferred doctor, Deprivation

## Abstract

**Background:**

From 2004 to 2009 there was almost a 12% rise in emergency admissions in England. This can be explained partly by an aging population and other socio-demographic characteristics, but much cannot be explained by these factors. We explored aspects of care, in addition to known demographic characteristics in general practice, that are associated with emergency admissions.

**Methods:**

A cross-sectional design employing hospital admission data from 76 general practices in Northamptonshire, England for 2006–08, including demographic data, quality and outcomes framework points and GP patient survey outcomes.

**Results:**

There were statistically significant associations between emergency admissions and age, gender, distance from hospital and proportion classified as white. There was also a statistically significant relationship between emergency admissions and being able to book an appointment with a preferred doctor; this relationship was stronger in less deprived communities.

**Conclusions:**

Enabling patients to book with a preferred doctor, particularly those in less deprived communities could have an impact on reducing emergency admissions. It is possible that being able to consult a preferred GP gives patient’s confidence to avoid an emergency admission or it facilitates consistent clinical management that helps prevent the need for admission. However the findings only explained some of the variation.

## Background

Between 2004 and 2009 there was almost a 12% rise in emergency admissions in the NHS in England. Approximately 35% of all admissions are classified as emergency admissions, costing approximately £11 billion a year [1]. An emergency admission has been defined as *an admission which is unpredictable and at short notice because of clinical need*[2]. Emergency admissions in England are described in Table 1.

**Table 1:** Emergency Admissions in England

**In England, patients may be admitted to hospital as emergencies if, when they fall acutely ill or are injured:**	
1 they or their carers take them to the emergency department of a hospital;	
2 or they are taken to an emergency department by an ambulance;	
3 or a general practitioner (GP; including out of-hours services) arranges emergency admission via an emergency department or directly to a hospital ward;	
4 or by other routes, for example, through an outpatient department if a patient attends a clinic when seriously ill.	

Adapted from Blunt, Bardsley & Dixon (2010).

Potential explanations for emergency admission rates include patient factors (e.g. morbidity, seasonal variation, socioeconomic factors), the service (e.g. type of care given to people at risk for admission), and data issues (e.g. changes in recording habits). Some of the increase can be explained by an aging population as admission rates have been greater in the over 75 s [3], and as much as 45% of the variance of practice emergency admissions has been reported to be explained by patient sociodemographic characteristics e.g. deprivation. Variation in admittance thresholds may also explain around 10% of the variance between different hospitals [4], but nevertheless, much of the increase in admissions cannot be explained by these factors alone.

National polices to reduce the trend in emergency admissions have included: accident and emergency four-hour waiting target, payment by results, community matron services, systems to identify patients with an increased risk of admission [5], and more recently financial penalties for readmissions within 30 days [6] and new quality and outcomes framework (QOF) emergency admissions indicators that encourage general practices to review emergency admissions [7]. These approaches on the whole have failed to control the rise in admissions, and recent guidance has suggested that small shifts in the proportion of patients that use primary care, rather than secondary care for urgent conditions, could have a large impact on emergency admissions [8]. Consequently, the role of primary care in reducing emergency admission rates has been recognised as potentially important [9]. Some variation in admissions can be explained by the size of the practice [10,11] and distance from hospital [11] although there may be no relationship between the type of population served, the organisation of primary care and admission rates [12,13]. Greater continuity in primary care has been associated with lower rates of hospitalisation [9,14], with access to a preferred GP being associated with reduced admissions [11].

With the introduction of the GP patient experience survey in 2007 [15] and the QoF in 2004 [16], practice level access and aspects of clinical performance can now be assessed. We undertook a cross-sectional study to investigate aspects of care (access and clinical performance), in addition to known demographic characteristics in general practice, that may be associated with emergency admissions.

## Methods

### Setting

The study draws on data from Northamptonshire, England, a county that has a population of 670,000 people. There are wide differences in socioeconomic status across the county [17]. There is one Primary Care Trust (PCT), NHS Northamptonshire, two general hospitals, both with emergency departments (ED), two minor injury units (cuts, strains, itches and strains) and 76 general practices.

### Admissions

We used anonymous emergency admission [2] data, which was obtained (with permission) from NHS Northamptonshire for two years (1st April 2006 to 31st March 2008) for all general practices in Northamptonshire to all hospitals. Maternity related admissions, admissions for patients registered with practices outside Northamptonshire and duplicates were excluded.

### Practice characteristics

Most (≈99%) of the population in England is registered with a GP [18,19]. GP practices usually have a team of medical, nursing and administrative staff. The GP patient survey was used to assess patient access [15] to general practice and is freely available online. The survey takes a sample of patients registered with each general practice and in 2007 and 2008 asked questions relating to: telephone access, an appointment within 2 days, ability to book an appointment in advance, with a particular doctor and satisfaction with opening hours (Table 2). We used these five aspects to assess access to the practice. The survey also collected information on the respondents’ ethnicity which was used to assess the proportion “white” or other within the practice.

**Table 2:** The quality and outcomes framework and access survey measures 2007 and 2008

**Access survey**	
Telephone access	In general, are you satisfied with how easy it is to get through to someone on the phone at your doctor's surgery
An appointment within 2 days	Think about the last time you tried to get an appointment with a doctor fairly quickly. Were you able to get the appointment on the same day or on the next 2 days the surgery was open
Able to book an appointment in advance	Last time you wanted to, were you able to get an appointment with a doctor more than 2 full days in advance
Able to make an appointment with a particular doctor.	Last time you wanted to, were you able to make an appointment with a particular doctor – even if it meant waiting longer
Satisfaction with opening hours	Over the last 6 months or so, were you satisfied with the hours your GP surgery was open
**Quality and outcomes framework (% achievement)**	
Clinical domain	80 indicators relating to 19 clinical areas (coronary heart disease, stroke, hypertension, diabetes, COPD, epilepsy, hypothyroidism, cancer, palliative care, mental health, asthma, dementia, depression, chronic kidney disease, atrial fibrillation, obesity, learning disabilities, smoking) worth up to 655 points.

Information on general practice performance from the publicly available QoF [16] was obtained for the corresponding years, 2006/07 and 2007/08. QoF is the pay and performance scheme set out in the national contract for GPs in England. The scheme offers financial rewards to practices according to how well (defined by indicators) they care for patients registered with them. Indicators cover four main components: clinical care, organisation of the practice, patient experience and additional services offered to patients. Total clinical care points were used to assess whether there was a relationship between practices of generally higher or lower reward for clinical care, access survey variables and admissions rate (Table 2).

Distance in kilometres from the practice to the nearest hospital within the county was calculated from a route planner [20], the practice postcode index of multiple deprivation 2007 (IMD) was used as an indicator of deprivation [21] and practice list size, age and sex structures were obtained from NHS Northamptonshire for years 2006/07 and 2007/08 for analysis.

### Statistical methods

Analysis was undertaken in STATA version 11.2 [22]. We present descriptive analysis of the emergency admissions to the two general hospitals from patients registered with the 76 practices within Northamptonshire carried out for each year separately, including the access survey and other data. Four general practices had missing patient access survey data for 2006/07 and data from these four practices for this year were excluded in the analysis. All 76 practices had complete data in 2007/08 and were included.

Negative binomial regression modeling was used to examine the association between numbers of emergency admissions from each practice in each year, and the practice level data on demographics, patient access and QoF [23]. This technique was used in order to allow for suspected over-dispersion of the data (i.e. a tendency for practices to have much greater differences in levels of acute admissions than would be expected by chance or the practice characteristics available for analysis). Robust estimates of the standard errors were calculated in order to allow for the expected similarity in admissions from the same practice in different years [24]. Modelling was carried out in three stages.

Stage one of the analysis included those variables assumed to have a major impact on hospital admissions, as indicated by previous studies [4,11,12,25]. These variables (Table 3) were: distance from hospital, IMD for the practice, proportion of the practice population of white ethnic group and proportion males. We used the proportion of people aged over 65 years, as there is a strong correlation with increasing age and increased morbidity in practices (Pearson correlation between the proportion aged >=65 years and the proportion on QoF practice disease coronary heart disease registers, 0.802, P<0.0001). Practice list size was used as the denominator for the number of admissions and therefore not used as a predictor. Deprivation quintiles were derived from IMD scores within Northamptonshire.

**Table 3:** Demographic Model - Emergency hospital admissions in terms of practice-level demographic data, derived using the BIC criterion

**Variable**	**Incidence Rate Ratio (IRR)**	**95% CI**	**P value (2-sided)**
Distance (per km)	0.9874	0.9762 to 0.9987	0.028
IMD (per unit)	1.0014	0.9971 to 1.0057	0.519
Interaction term:			
Distance (per km) × IMD (per unit)	1.0009	1.0004 to 1.0014	0.001
Age65≥ years (per 1%)	1.0224	1.0095 to 1.0355	0.001
Males (per 1%)	1.1535	1.0899 to 1.2209	<0.001
White population (per 1%)	1.0990	1.0520 to 1.1481	<0.001
Interaction term:			
Males (per 1%) × White pop (per 1%)	0.9981	0.9973 to 0.9989	<0.001

BIC (Bayesian information criterion) = 1865.306.

IRR less than 1.0 represent decreases and IRR greater than 1.0 represent increases in the count.

Stage two of the analysis examined the possible association between patient survey characteristics and hospital admissions, while accounting for the variables included in Stage one. The five measures of continuity and access from the patient survey (Table 2) were considered for inclusion in the model one at a time, considering each variable independently. Non-significant stage two variables were removed in order to determine which of the stage two variables were significant multivariable predictors of the admissions rate [4,10,12]. The interaction of each patient survey variable with each demographic variable was then examined in the model, one at a time. The degree to which various candidate statistical models fitted the observed data was compared using the Schwarz Bayesian Information Criterion (BIC).

Stage three of the analysis used total QoF clinical points (as a global measure of the achievement of practices with respect to the financial incentive for clinical care given) to explore the effect of practices’ success in this domain on the relationship between access variables and admissions. P values were two-sided and under 0.05 were considered statistically significant.

## Results

There were 57,954 admissions in 2006/07 and 57,398 admissions for 2007/08, (total 115,252). Just of 84% over all emergency admissions were to the two hospitals within Northamptonshire. Table 4 shows the descriptive statistics used in the analysis for each year and combined years (median of the 148 separate figures for each practice-year). There was a median 580 (mean 759) emergency admissions per practice per year with a wide inter-quartile range (367 to 1132) and median rate of 87 per 1,000 patients. IMD scores show that on average Northamptonshire is in the middle quintile of deprivation across England, although having practices in the 20% most deprived (IMD ≥34.44) and 20% least deprived (IMD ≤8.31) nationally.

**Table 4:** Descriptive statistics for predictors used in the statistical models

	**2006/7**		**2007/8**^**+**^	
	**72 practices**		**76 practices**	
	**median**	** IQR**	**median**	** IQR**
Total QoF clinical points	652	635 - 654	652	649 – 654
% Satisfied with phone access	89	78 - 94	88	80 – 95
% Able to book 2 days ahead	72	51 - 87	77	63 – 87
% Able to get an appt in 48 hours	88	80 - 94	90	82 – 94
% Able to book with a preferred GP	87	80 - 92	87	80 – 92
% Satisfied with opening hours	86	81 - 89	82	77 – 86
Distance from hospital (km)	8.9	3.5 – 15.3	10.2	3.6 – 15.3
% of practice male	50	50 - 51	50	50 – 51
Age (% of practice patients aged 65+)	14	12 - 16	14	12 – 16
Practice deprivation score (IMD)	19	1 – 28	19	1 – 29
% of practice white ethnicity	92	87 - 95	90	85 – 94
Practice list size	7505	4454 - 11249	7561	4501 - 11542
Practice’s acute admissions per year	589	376 - 1118	580	359 – 1132
Acute admissions per 1000 patients	85	75 - 103	89	75 – 102

^+^ including the 4 practices assessed only in 2007/8.

IQR = inter-quartile range.

In stage one of the analysis, age, proportion white and proportion males and distance from ED were significant demographic variables to predict an emergency admission (Table 3). The table gives IRR of less than 1 for increasing distance from ED suggesting that increasing distance is associated with lower admission rates (Table 3). Though, there was a interaction between distance and deprivation, with increasing deprivation, admissions increased with increased distance, whereas in less deprived practices, increasing distance reduces admissions. Additionally each increase of 1% of the practice population being over 65 years old, was associated with a relative increase of 2.2% of acute admissions (P=0.001). There was a negative interaction between ethnicity and gender.

Stage two of the model found that only the ability to book an appointment with a preferred doctor was significant (P=0.020) (Table 5). No further access-survey variables, or interactions of these with a demographic variable, improved the BIC criterion when added to that model.

**Table 5:** Uni-variable analyses: The effect of each Access Survey characteristic when included, one at a time, in the Demographic model

**Access Survey Variable**	**Incidence Rate Ratio (IRR)**	**95% CI**	**P value (2-sided)**
Satisfaction with phone access (per 1%)	0.9985	0.9952 to 1.0019	0.386
Booking within 48 hours (per 1%)	1.0013	0.9966 to 1.0006	0.589
Booking >2 days in advance (per 1%)	0.9992	0.9971 to 1.0013	0.468
Booking with preferred doctor (per 1%)	0.9942	0.9894 to 0.9991	0.020
Satisfaction with opening hours (per 1%)	0.9994	0.9926 to 1.0063	0.866

In the final stage of the model, the QoF clinical points were non-significant (P=0.829). Although the interaction between the effects of ethnicity and booking with a preferred doctor on the admission rate was significant (P=0.006), we included in the final model the interaction between deprivation and the ability to book with a preferred doctor (P=0.043), as the deprivation variable had a greater reliability than the ethnicity data, which had been derived from respondents to the access survey (Table 6). The interaction between the percent able to book appointment with preferred GP and deprivation on emergency admissions is shown in Table 7.

**Table 6:** Access Survey and Demographic Model

**Variable**	**Incidence Rate Ratio (IRR)**	**95% CI**	**P value (2-sided)**
Distance (per km)	0.9904	0.9811 to 0.9998	0.046
IMD (per unit)	0.9680	0.9351 to 1.0020	0.065
Interaction term:			
Distance (per km) × IMD (per unit)	1.0009	1.0004 to 1.0013	0.0005
Age65≥ years (per 1%)	1.0228	1.0090 to 1.0367	0.001
Males (per 1%)	1.1379	1.0720 to 1.2078	<0.001
White population (per 1%)	1.0874	1.0400 to 1.1369	<0.001
Interaction term:			
Males (per 1%) × White pop (per 1%)	0.9983	0.9975 to 0.9992	0.0001
% Able to book with a preferred GP (per 1%)	0.9859	0.9766 to 0.9953	0.004
Interaction term:			
% Able to book with a preferred GP (per 1%) × IMD (per unit)	1.0004	1.0000 to 1.0008	0.043
QoF clinical points (per 10 points)	0.9987	0.9968 to 1.0107	0.829

Emergency hospital admissions in terms of practice-level Access Survey, QoF and demographic data, derived using the BIC criterion.

BIC (Bayesian information criterion) = 1864.690.

**Table 7:** Interpretation of the interaction between the association of deprivation and booking with a preferred doctor with emergency admissions, from the Access Survey and Demographic model

	**For *each *1% absolute increase in %Able to book with a preferred GP**	**For the ‘typical’ (median) practice with 580 EAs per year – the corresponding decrease in EAs**	**Median value of ‘% Able to book with a preferred GP’ for each IMD Quintile**
Deprivation quintile 1 (typically 6 IMD points) - Least deprived	decrease of 1.2% in EAs	decrease of 6.8 EAs	87%
Deprivation quintile 2 (typically 13 IMD points)	decrease of 0.9% in EAs	decrease of 5.2 EAs	87%
Deprivation quintile 3 (typically 20 IMD points)	decrease of 0.6% in EAs	decrease of 3.6 EAs	86%
Deprivation quintile 4 (typically 25 IMD points)	decrease of 0.4% in EAs	decrease of 2.4 EAs	89%
Deprivation quintile 5 (typically 40 IMD points) - Most deprived	increase of 0.2% in EAs	increase of 1.1 EAs	87%

EA = Emergency Admission.

## Discussion

### Main finding

Potentially, the most interesting finding of this exploratory study may be the association between seeing a preferred doctor, emergency admissions and the interaction with deprivation. This study also found statistically significant associations between emergency admissions and age, proportion males, distance from hospital and proportion of the practice population who were classified as white, although all of these findings were relatively small. The relationship found between deprivation (IMD) and ability to see a preferred doctor was different between those practices with lower IMD (practices in less deprived locations) and higher IMD (practices in more deprived locations). The relationship was stronger in those practices in less deprived locations and negligible generally in the practices in the most deprived areas. For example in a typical least deprived practice and every 1% increase in seeing a preferred doctor relates to a decrease in almost 7 emergency admissions compared to a small increase of just over 1 in a typical most deprived practice (Table 7).

### What is already known on this topic

Access is believed to reduce the use of secondary care, which has influenced national initiatives to improve access [26,27]. Access is seen as key to securing relationship continuity between a GP and patient. Continuity requires ready access to the GP [28]. This relationship has been described as ‘inextricably intertwined’ [29]. Continuity has been defined as from the patient perspective as a doctor to whom they want to consult [30]. Being able to see a preferred GP can be more important than quickness of appointment, however, when a problem is ongoing and of high emotional impact, patients show preference to seeing a familiar clinician [31]. This suggests continuity may be more important than immediate access in those situations. The finding that continuity may impact on emergency admissions has been found elsewhere and importantly continuity of care has been shown to be declining [32]. It is possible that the relationship (the same provider or providers consulted for most problems) facilitates management and informational continuity, although record systems and effective co-ordination may achieve, at least in part, management and informational continuity as well.

Building relationships between patients and practitioners takes time, over a number of interactions. In contrast, in single encounters with a GP, the relationship is more vulnerable and some aspects of patients’ healthcare needs could remain unmet [33]. It is possible that being able to consult a preferred GP gives patients the confidence to avoid an admission or that it facilitates consistent clinical management that helps to prevent the need for admission. However, patients that have changes in usual care, identify more unmet healthcare needs than those with no usual care [34]. This suggests that once a healthcare need is identified, continuity of care can be important for patients.

### What this study adds

Our study confirms the findings of other studies that show an association between deprivation, age, proportion male, ethnicity and continuity in general practice in relation to emergency admission rates [9,11]. The study adds the novel, but plausible hypothesis that more people able to book with a specific GP is on average associated with a decrease in emergency admissions in the least deprived quintile of practices, yet a negligible increase in the most deprived quintile. This finding is important because it indicates that initiatives to reduce admissions amongst deprived and more affluent populations may be different. The explanation for the reduced effect of continuity in deprived populations is not clear.

Different social groupings have previously been shown to have different perceptions of care i.e. seeing a preferred doctor [35], providing one potential explanation for the effect of deprivation on the association between continuity and admission rates, but it could be that the most deprived populations have such a high level of disease that continuity of care has no effect on emergency admissions. Alternately the doctor patient relationship among more deprived populations, although improved by continuity, does not appear to affect their behaviour in the use of emergency services.

The relationship found could be a combined factor of differences in deprivation between urban and rural populations and differences in age structures (i.e. older, less deprived communities tend to live in rural communities [17]. The relationship that rural residents on higher incomes travel to urban areas with hospitals more frequently has been found [36], yet other studies have shown that the closer you live to hospital the higher the admission rate [37]. The relationship and interaction with deprivation would need to be tested further, for example if the findings hold true at a national level.

A similar study in Leicestershire [11] found that being able to see a preferred GP, age, and ethnicity were associated with emergency admissions. However, the study found that there were associations between practice list size, and the closer to a hospital the increased association with emergency admission. These differences may be due to differences in healthcare facilities available i.e. hospital location and access, or differences in population demographics i.e. high Asian ethnicity in Leicester city or differences in how patient characteristics were determined in the model i.e. age, ethnicity and deprivation. Our findings may also be influenced by the particular geography of Northamptonshire and not be applicable to other settings. For example, the county includes a town with a relatively deprived population (Corby) set at some distance from the county’s acute hospitals.

Nevertheless, our findings confirm other studies in which age, ethnicity, gender and deprivation were associated with increased hospital admission, and support initiatives to reduce admissions in those communities. The study does not provide evidence of effectiveness of interventions to reduce admission rates, but suggests that general practice appears to have a part to play, particularly in patient groups at higher risk of admission and those from different socioeconomic backgrounds. Yet further research is required to establish the impact of interventions to improve continuity of care from general practice in these groups.

### Limitations of this study

There are several limitations of our study. This is an ecological study and our findings may not be applicable to individuals and this is a study showing associations, not causal relationships. There may be one or more variables that were omitted in our analysis that may explain admission rates, as the associations found only accounted for a small proportion of the variation. Better health has been associated with non-medical determinants of health i.e. deprivation, therefore healthcare performance may be less likely to be related to health overall [38].

QoF points represent the financial rewards earned by practices and reflect the effectiveness of practices in responding to the incentive scheme. Better organised practices are more likely to achieve maximum points than less well organised practices, however this may not impact on the care provided by the practice. With the lack of variation in the QoF clinical points gained from practices and with low thresholds to obtain full points, QoF may not be sensitive to the full range of effectiveness of practices.

The results from this study are only for two years analysis and from one county, and may not be applicable to other localities. Sixteen percent of admissions went to other hospitals outside Northamptonshire which we did not measure distance to the specific hospital, which may introduce some bias. The GP patient survey comprises patients’ subjective reports of access, and the survey findings are therefore likely to reflect patient expectations and response sets rather than objective measurement of access alone, additionally the association between deprivation and booking with a specific doctor, although significant at the 5% level was relatively weak. Furthermore, the survey only addressed certain aspects of access [4]. The lack of absolute proportion non-white, the use of deprivation and distance to the closest hospital from the practice, rather than the individual patient may also have an impact on the results. These need to be considered in the associations found in our results.

## Conclusions

Our study found that those practices with more patients who were able to book with a preferred doctor had lower emergency admissions and that this relationship was stronger in less deprived communities. It is possible that being able to consult a preferred GP gives patient’s confidence to avoid an emergency admission or it facilitates consistent clinical management that helps prevent the need for admission. Those planning primary care services, and those providing them, should avoid policies that reduce continuity of care. Further research is needed to understand the reasons why this finding did not predict admission rates in the most deprived populations and if they are replicable nationally.

## Competing interest

The authors declare that they have no competing interests.

## Authors' contributions

All authors were involved in developing and implementing the study. SG acquired the data and developed the analysis protocol, interpreted the findings and drafted the paper. NT undertook the analysis and helped draft the manuscript. SR and RB conceptualised the study and participated in its design of the study and coordination and helped draft the manuscript. All authors read and approved the final version of the manuscript.

## Pre-publication history

The pre-publication history for this paper can be accessed here:

http://www.biomedcentral.com/1472-6963/13/11/prepub
